# Level of health technology assessment process-related skills among doctors in Croatia: a cross-sectional survey study

**DOI:** 10.1017/S0266462326103572

**Published:** 2026-02-25

**Authors:** Miro Vuković, Mirjana Huić, Ljubo Znaor, Željko Krznarić, Ana Marušić

**Affiliations:** 1 Center for Evidence-Based Medicine, University of Split School of Medicine, Croatia; 2 HTA/EBM Center, Croatia; 3 University Hospital of Split, Croatia; 4 Croatian Medical Association, Croatia

**Keywords:** Health technology assessment (HTA), EU HTA Regulation (HTAR), HTA capacity building, Clinical experts, Evidence-based medicine, Central and Eastern Europe

## Abstract

**Introduction:**

The European Union’s Health Technology Assessment Regulation (HTAR) and its implementing acts foresee various forms of clinician involvement, such as joint clinical assessment or Joint Scientific Consultation. However, considering the varying preparedness levels for HTAR, as well as the understanding of the health technology assessment (HTA) principles and processes, this study aimed to evaluate the levels of HTA-related skills among medical and dental doctors in Croatia.

**Methods:**

A cross-sectional survey study was conducted among medical and dental medicine doctors in Croatia. The survey recorded respondents’ relevant experience with HTA processes along with skill levels across the entire HTA process, mainly for acting as individual clinical experts or on behalf of their professional organizations, as well as potential HTA doers. Skill levels were evaluated using a 5-point scale (1 – *no knowledge* to 5 – *full expertise*).

**Results:**

Among the 376 respondents included, only 6.1 percent had previous involvement in HTA, and 2.2 percent were familiar with HTAR. Related to the HTA process, the highest scores were observed in the understanding of key concepts and results of searching for studies, critical appraisal, study synthesis preparation, and ethics. The lowest scores were recorded in health economics, evidence grading, qualitative synthesis, and public/patient involvement. Respondents with prior research experience and those who reported frequent research use had significantly higher HTA skill scores.

**Conclusions:**

A significant gap in HTA-related skills highlights the need for targeted professional development programs and long-term educational reforms to build the capacity for various modes of involvement in HTA processes and their implementation.

## Introduction

In response to the growing need for evidence-based decision-making, the continuous advancement of health technologies combined with limited healthcare budgets has driven the establishment and expansion of health technology assessment (HTA) as an essential tool for informed policy and practice (1). HTA focuses on the added value of health technology compared to technologies to be replaced (2), providing input to decision-making in policy and practice, and ensuring value for money (3). As a health policy tool that gives healthcare the necessary transparency (4), HTA can drive positive changes in the financial sustainability and administrative efficiency of the healthcare system (5;6). It can also improve the quality of healthcare for patients by informing national evidence-based decision-making and summarizing the best available clinical and nonclinical evidence. The benefits of HTA in enhancing the responsiveness of health systems to patients’ needs and promoting equity in healthcare have been well-documented (7;8). Additionally, HTA contributes to strengthening independent research and enhancing the overall quality of scientific evidence (9). These outcomes, in turn, support the development of more robust clinical practice guidelines, help identify research gaps, and inform national decisions on including medicines in reimbursement lists (10–12).

In the European Union (EU), the Health Technology Assessment Regulation (HTAR) represents the first step toward harmonizing joint clinical assessment (JCA) of a health technology across EU member states (2). Implemented since January 2025, HTAR, among other benefits, helps achieve a high level of health protection for patients and users while ensuring the smooth functioning of the internal market regarding medicinal products, medical devices, and in vitro diagnostic medical devices. At the same time, the HTAR establishes a framework to support sustainable cooperation among Member States and defines the structure of the JCA. The JCA is based on the scientific aspects of the clinical domains of HTA, including the description of the health problem addressed by the health technology and the current use of other health technologies addressing that health problem, the description and technical characterization of the health technology, the relative clinical effectiveness, and the relative safety of the health technology. At the same time, methodological guidance for their implementation is provided by the HTAR Subgroup for the development of methodological and procedural guidance (13). The HTAR and its implementing acts also establish a transparent process and general principles for conducting Joint Scientific Consultations (JSCs) and identifying emerging health technologies (horizon scanning). It further ensures the appropriate involvement of various stakeholders – such as patients, clinical experts, and their respective organizations – in joint work.

Bearing in mind that HTAR must be implemented in various settings and healthcare systems, several limitations to its national implementation exist. The main limitations relate to limited funding and insufficient training opportunities for HTA professionals, which reduce the capacity to produce timely and high-quality HTA reports and engage effectively with policymakers (14). Since the adoption of HTAR, several EU-level initiatives have been launched to address this gap, including European Health and Digital Executive Agency’s 2024 call for tender (€ 35 million budget) for JCAs, JSC, and the HTA Capacity Building (HAG INSIGHT) project, which aims to provide structured training for assessors (15;16). Furthermore, considering that a well-conducted HTA requires multidisciplinary teams and adaptable human resources, the need for training programs to ensure trained personnel is even more evident (17). Patients and clinical experts, as well as their respective organizations, also require continuous education for their timely and practical involvement in joint work and in national HTA processes. Two EU projects addressing the capacity-building needs of patients and patient organizations were European Capacity Building for Patients (EUCAPA, completed) and HTA4Patients (currently ongoing) (18;19). Unfortunately, there are no current projects for clinical experts and their respective professional organizations.

These limitations are particularly evident in Central and Eastern European (CEE) countries. Many health systems in these countries face restricted financial resources for adopting innovative and often costly health technologies. This situation increases the importance of producing timely and high-quality HTA reports to guide efficient resource allocation. However, the region continues to face a shortage of trained HTA professionals – especially health economists – who must operate within limited institutional budgets (20). Furthermore, the pricing/reimbursement rationale of new healthcare technologies (especially pharmaceuticals) in Europe is primarily driven by early market entry strategies of pharmaceutical companies seeking to establish price benchmarks in large countries (21;22). Moreover, many CEE countries still lack a clear roadmap for systematic HTA implementation. Comparisons with high-income and resource-rich countries are often not directly applicable. CEE health systems cannot allocate comparable financial and human resources levels to support evidence-based policy-making. (23).

In Croatia, preparatory work for implementing the HTAR has been initiated under the coordination of the Ministry of Health, which assumed the responsibility for HTA from the Agency for Quality and Accreditation in Health Care in 2019 (24). The Ministry currently acts as a member of the HTA Coordination Group, in both formats: for medicinal products and medical devices, as well as a member of four subgroups (for JCA, JSC, the identification of emerging health technologies, and the development of methodological and procedural guidance). A national working group for HTAR implementation was established in 2023 to align the national legal framework and national HTA process with HTAR. The integration of JCA outcomes into national decision-making is expected to follow a “European assessment – national assessment – national appraisal” model, whereby clinical evidence jointly assessed at the EU level will be used in the national HTA reports and nonclinical domains. Such national HTA reports will then inform reimbursement and pricing decisions made by Croatian authorities (25;26). Although moderate progress has been achieved, including the establishment of national coordination structures and partial engagement in EU-level initiatives, a significant amount of work remains to be done to ensure the full operationalization of HTAR processes and the sustainable integration of JCAs within national HTA processes and the Croatian healthcare system.

Given these challenges, our study focused on identifying and evaluating key skills and training needs of medical and dental medicine doctors, as they are envisioned as both potential HTA doers and individual clinical experts/stakeholders in the context of HTAR in Croatia. While a few doctors will be expected to have the role of HTA doers, much more are expected to understand the HTA process and contribute to various areas of joint work and other steps of the HTA process, from early horizon scanning and PICO (Patient-Intervention-Comparison-Outcome) framework development for JCA scope to participation in national assessments and further appraisal processes and implementation of HTA results in practice. By understanding the essential capabilities necessary for conducting HTA and for effective stakeholder involvement, both at the EU level and in national processes, this work aims to inform targeted capacity-building initiatives and long-term strategies to ensure successful implementation of HTAR in Croatia.

## Methods

### Study design

In a quantitative cross-sectional study conducted in 2023, we surveyed medical/dental doctors who work in academic health institutions or are members of the Croatian Medical Association, the professional organization of medical doctors.

### Croatian healthcare setting

All hospitals in Croatia are operated by the government and classified into five categories (20): clinical hospital centers, clinical hospitals, clinics, general hospitals, and specialist hospitals. These institutions’ descriptions are retained to provide context for the study setting and the rationale for choosing university hospitals for participant recruitment – they represent the primary academic and clinical environments in which potential HTA stakeholders and future HTA practitioners are likely to work.

The Croatian Medical Association is an umbrella organization for all professional medical societies in Croatia dedicated to improving professional and scientific work, public health protection, member interests, and medical ethics (27). The Association’s organizational components include 26 subsidiaries in major Croatian towns and 126 professional societies and sections dedicated to various medical specialities or topics. Participants from the Croatian Medical Association were also included, as they contribute to the creation of Croatian clinical practice guidelines (28). Previous literature highlights the involvement of medical societies as a critical success factor for EU HTA (29).

The implementation of HTA in Croatia started in 2009, with the work of the Department for Development, Research, and HTA within the then newly established Agency of Quality and Accreditation in Health Care (24). In 2019, the Ministry of Health in Croatia assumed the responsibilities of the Agency (30). HTA has never been mandatory either before or after this transition; it represented an optional component in decision-making on health technologies. Some HTA reports on medicinal products and medical devices have been produced through collaboration at the EU level (within EUnetHTA Joint Action 1–3 projects). After completing joint processes, reports were translated and adapted for national use and made publicly available through the Croatian Ministry of Health website (31). The EUnetHTA originals were always attached to such Croatian HTA reports, with links to the respective EUnetHTA site.

### Participants

The target population for the study was practicing academic doctors who could primarily serve as potential individual clinical experts or members of health professional organizations but also as potential HTA doers, participating in JCA, JSC, identification of emerging health technologies, and voluntary cooperation at the EU level, as well as in national HTA processes (32). Eligible participants were doctors of medicine and dental medicine from the University Hospital Centers in Zagreb and Split – the two largest hospital centers in Croatia and national hubs of clinical excellence. Dental doctors were included because they are formally recognized as part of the same professional body within the Croatian healthcare system and the Croatian Medical Association. They may participate in similar evidence-based and policy-oriented professional activities. Including both groups provided a more comprehensive assessment of clinical professionals’ skills related to HTA.

### Sampling

A nonprobability convenience sampling method was used. An open invitation to participate was circulated to doctors through the hospital’s electronic networks and the Association’s mailing lists. In addition, printed questionnaires were distributed directly by study collaborators to ensure inclusion of participants who used institutional e-mail less frequently.

### Sample size calculation

Using the digital registry of the Croatian Medical Chamber, we calculated that the number of physicians employed at the University Hospital Centers in Zagreb and Split was 2,815 at the beginning of our study. Based on a 95 percent confidence level and a 5 percent confidence interval, the minimum required sample size was calculated to be 339 respondents.

### Survey instrument

We used a modified version of a previously published questionnaire to assess experience and skills necessary for four HTAR areas of joint work and the national HTA process, in which they could be primarily involved as individual clinical experts or on behalf of their respective organizations but also as an HTA doer (33). The modified version was extensively revised to align with the study objectives and Croatian context.

The questionnaire used comprised the following parts:

- An introduction explaining key terminology;
- Questions on demographic information;
- Items on past experience in HTA, evidence synthesis, and economic evaluation (relevant to act as an HTA doer);
- Items assessing skills required in the HTA process (relevant to act as an individual clinical expert or member of health professional organizations);
- Items on experience related to EU joint work (relevant to act as individual clinical expert or member of health professional organizations or HTA doer);
- Items addressing perceived training needs for national and EU-level HTA (relevant to act as an individual clinical expert or member of health professional organizations).

The full version of the questionnaire is available as Supplementary File 1.

Although the questionnaire used in this study was not validated, we verified internal consistency using Cronbach’s alpha to confirm whether groups of related questions consistently capture the same construct within each skill domain. Results of the part about Items addressing perceived training needs for national and EU-level HTA are not presented here since a large proportion of responses were incomplete or misunderstood and hence were excluded from the analysis.

### Data collection

We collected data on age, gender, education, field of work, years of professional experience, previous HTA involvement, and research experience.

The level of understanding of steps and results of the HTA process, mainly relevant to act as an individual clinical expert or member of health professional organizations, was calculated as an aggregated score from answers given in the “Questions regarding skills needed in the HTA process” section, which contained items grouped into 10 domains:

1. Clinical effectiveness and safety (understanding the PICO acronym; writing a structured research question according to the PICO acronym; formulating the key words for the research question);
2. Searching for the studies (understanding the importance of electronic sources and databases to search for evidence; understanding the importance of gray literature: ongoing studies and unpublished data sources; understanding the importance of other search approaches (hand searching and citation snowballing));
3. Critical appraisal skills (identifying validated tools to appraise scientific literature critically; recognizing the most critical domains to appraise in the different study designs; understanding of the procedure through which critical appraisal should be undertaken);
4. Summarizing study characteristics and preparing for synthesis (understanding the summary of the characteristics of each study; determining comparability across studies (i.e., clinical, statistical, and methodological heterogeneity); understanding the relevant comparisons from the included studies);
5. Qualitative evidence synthesis (understanding the use of qualitative evidence synthesis in HTA; understanding the methods for qualitative evidence synthesis: thematic synthesis, framework synthesis, and meta-ethnography; understanding the importance of balanced description and interpretation);
6. Grading the certainty of evidence (understanding the summary of findings (SoF) tables or evidence profiles; understanding the ranking of the outcomes for SoF tables or evidence profiles; understanding the various approaches for assessing the certainty of a body of evidence);
7. Understanding key concepts in data synthesis and analysis (results from meta-analysis; results from meta-regression; heterogeneity; results from subgroup and sensitivity analysis; results from narrative synthesis when a meta-analysis is not possible (e.g., logical categories); results from network meta-analysis; principles of a network meta-analysis (transitivity or evidence network));
8. Ethics (understanding the ethical issues concerning technologies; understanding the bioethical issues and concepts (self-determination, privacy, informed consent, etc.));
9. Public and patient involvement (understanding the importance of public and patient engagement (PPI) in HTA; understanding the mode of engagement; understanding the process of identification and recruitment of those most affected by the decision; understanding the input from PPI);
10. Health economics (understanding the objectives of economic evaluation of health interventions (e.g., using cost-effectiveness analysis and cost-utility analysis); understanding the results of economic evaluation of health interventions (e.g., cost-effectiveness analysis and cost-utility analysis); understanding the results of budget impact analysis).

Each domain had two to seven questions rated on a five-point scale:

1 – No knowledge of this topic;

2 – I have heard of this topic, but I do not feel confident doing it;

3 – Slightly confident to do it;

4 – I am confident to do it;

5 – I have full expertise in this topic.

Respondents with missing answers within a domain were excluded from that domain’s analysis.

### Statistical analysis

Categorical data were presented as frequencies and percentages. In contrast, numerical data were reported as medians with interquartile ranges or means ± standard deviations, depending on the data distribution, which was assessed using the Shapiro–Wilk test.

Cronbach’s alpha coefficients were calculated to assess internal consistency within each skills domain, indicating how reliably the grouped questions measured the intended construct.

To explore relationships between demographic factors and skill scores, Kruskal–Wallis and Mann–Whitney tests were performed considering the following variables:

1. Age (<30, 31-40, 41-50, 51-60, >60 years);
2. Highest level of education achieved (MD/DMD, MSc, PhD, other);
3. Hospital of employment (University Hospital Split, University Hospital Zagreb, Other);
4. Working experience (<5 years, 5–10 years, 11–15 years, 16–20 years, more than 20 years of experience);
5. Experience in working in a decision-making body in healthcare (yes/no);
6. Previous experience in conducting research (yes/no);
7. Current international collaboration (yes or no);
8. Level of research use in everyday work (lowest level: I do not use research or use it very rarely, highest level: I work as a researcher; “use of research” in this context refers to reading and applying peer-reviewed scientific publications in daily professional work).

All statistical analyses were performed using MedCalc Statistical Software version 22.023.0 (MedCalc Software bv, Ostend, Belgium; https://www.medcalc.org; 2020). The significance level was set at *p* < 0.05, following the conventional threshold for exploratory analyses.

## Results

### Demographic characteristics

A total of 383 respondents completed the questionnaire. Responses from seven participants were excluded due to substantial missing data (e.g., key demographic data or most of the questionnaire) (Supplementary Table 2). Most of the 376 participants were female and aged between 31 and 40 years. About a third of the participants also had a scientific doctoral degree (PhD). Survey respondents represented a broad range of specialities without a single dominant field. Details of respondents’ fields of expertise are presented in Supplementary Table 3.

### Professional experience

Only about a third of the participants reported ongoing international research or professional collaboration, and approximately a fifth had participated in the work of a decision-making body. Most of the participants used published research articles in peer-reviewed journals occasionally, while the majority of them had at least some level of experience in conducting scientific research (Table 1).

**Table 1.** Clinical experts past experience* in health technology assessment (HTA) processes

	*N* (%)
Current international collaboration (*n* = 374)	
Yes	102 (27.3%)
No	272 (72.7%)
Have you ever participated in the work of a decision-making body in healthcare? (*n* = 374)	
Yes	82 (21.9%)
No	292 (78.1%)
Do you have experience in conducting scientific research? (*n* = 368)	
Yes	218 (59.2%)
No	150 (40.8%)
Use of research in everyday work (*n* = 366)	
I do not use research or use it very rarely	46 (12.6%)
Use research from time to time	169 (46.2%)
Use research in everyday work	119 (32.5%)
I work as a researcher	32 (8.7%)

* The number in a bracket next to each item indicates the number of valid responses for each demographic question.

When analyzing the previous evidence-synthesis experience, quality appraisal, and economic analysis, relevant to working as a possible HTA doer, most participants (*n* = 252; 67 percent) had not previously contributed to literature reviews or clinical practice guidelines. Only 9 percent had worked on economic evaluations, most commonly cost-effectiveness analyses. A detailed description of the respondents’ experience in evidence synthesis is presented in the Supplementary Table 4.

### Experience in the HTA process or other HTAR areas of joint work

Only a small proportion of participants had ever been directly involved in an HTA process, while a somewhat large group had used HTA results (Table 2).

**Table 2.** Clinical experts’ past experience* in health technology assessment (HTA) processes or different areas of joint work**

	*N* (%)
Have you ever been involved in the HTA process? (*n* = 360)	
Yes	22 (6.1%)
No	338 (93.9%)
Have you ever used the results of an HTA? (*n* = 349)	
Yes	50 (14.3%)
No	299 (85.7%)
Have you ever undertaken a systematic review or other type of evidence synthesis? (*n* = 343)	
Yes	139 (40.5%)
No	204 (59.5%)
Have you ever undertaken an economic evaluation? (*n* = 330)	
Yes	29 (8.8%)
No	301 (91.2%)
Have you ever used the results of an economic evaluation? (*n* = 319)	
Yes	34 (10.7%)
No	285 (89.3%)

* The number in a bracket next to each item indicates the number of valid responses for each demographic question.

** Horizon scanning, Scientific consultation processes, or Voluntary collaboration.

Respondents demonstrated little experience in economic evaluation. Familiarity with the EU HTAR was minimal: 64 percent of respondents reported being unfamiliar with it, 34 percent had only heard of it, and just 2 percent were familiar with its details.

Experience with EU joint work was also limited: 3 percent had participated in a Joint HTA, 8 percent in JSC, and 5 percent in horizon scanning. Almost none had been involved in preparatory phases for the implementation of HTAR at either the EU or national level (Table 3).

**Table 3.** Clinical experts’ experience* in work related to health technology assessment (HTA) process or different areas of joint work**

	*N* (%)
How familiar are you with the EU HTA Regulation (*n* = 237)	
Not at all	175 (64.1%)
Heard of it	92 (33.7%)
Familiar with the details	6 (2.2%)
Do you have previous experience as a clinical expert/member of health professional organizations in joint HTA? (*n* = 272)	
Yes	9 (3.3%)
No	263 (96.7%)
Do you have previous experience in Joint Scientific Consultation? (*n* = 271)	
Yes	23 (8.5%)
No	248 (91.5%)
Do you have experience in horizon scanning? (*n* = 271)	
Yes	15 (5.5%)
No	256 (94.5%)
Are you envisaging being involved as a clinical expert/member of health professional organizations within voluntary collaboration on HTA? (*n* = 271)	
Yes	28 (10.3%)
No	243 (89.7%)
Are you involved in the preparatory phase related to the EU HTA Regulation? (*n* = 270)	
Yes	3 (1.1%)
No	267 (98.9%)
Are you involved in the preparatory phase at the national level? (*n* = 268)	
Yes	6 (2.2%)
No	262 (97.8%)
Are you aware of any published EUnetHTA21 documents? (*n* = 270)	
Yes	10 (3.7%)
No	260 (96.3%)
Are you involved in any activities related to EU support for HTA? (*n* = 271)	
Yes	4 (1.5%)
No	267 (98.5%)

* The number in a bracket next to each item indicates the number of valid responses for each demographic question.

** Horizon scanning or scientific consultation processes.

### Skills needed in the HTA process, to act as an individual clinical expert or a member of health professional organizations

Aggregate results by questionnaire section are presented in Table 4. Cronbach’s alpha coefficients ranged from 0.903 to 0.972 (Supplementary Table 5), confirming high internal consistency.

**Table 4.** Clinical experts’ reported skills required for the health technology assessment process

Domain	No	Score range	Median score (IQR)	% Maximal score^1^
1. Clinical effectiveness and safety	271	3–15	7.0 (3.0–11.0)	47%
2. Searching for the studies	273	3–15	9.0 (6.0–11.0)	60%
3. Critical appraisal skills	272	3–15	9.0 (6.0–11.0)	60%
4. Summarizing study characteristics and preparing for synthesis	272	3–15	9.0 (6.0–11.0)	60%
5. Qualitative evidence synthesis	272	3–15	6.0 (3.0–8.0)	40%
6. Grading the certainty of evidence	272	3–15	6.0 (3.0–9.0)	40%
7. Understanding key concepts in data synthesis and analysis	273	7–35	18.0 (11.0–22.0)	51%
8. Ethics	272	2–10	6.0 (5.0–8.0)	60%
9. Public and patient involvement	274	4–20	8.0 (4.0–12.0)	40%
10. Health economics	274	3–15	6.0 (3.0–9.0)	40%
Total score	268	34–170	86.0 (62.5–103.5)	51%

1 Given that different domains have different numbers of questions (and subsequently, different maximum numbers of points, this percentage has been defined as a proportion of the median number of points achieved and the maximum number of possible points.

The highest median scores were recorded for the following domains and their corresponding items: searching for studies, critical appraisal skills, summarizing study characteristics, preparing for synthesis, and ethics. The lowest scores were obtained for domains related to qualitative evidence synthesis, grading the certainty of evidence, health economics, and public and patient involvement.

Sections with the highest scores had a median of 60 percent of the maximum possible points, whereas those with the lowest had 40 percent. This percentage is the proportion of the points achieved to the maximum possible points for each given domain. Given that different domains have varying numbers of questions (and consequently, different maximum point totals), this approach facilitates the comparison of skill levels across various domains.

### Factors influencing the total questionnaire score

**Figure 1.**
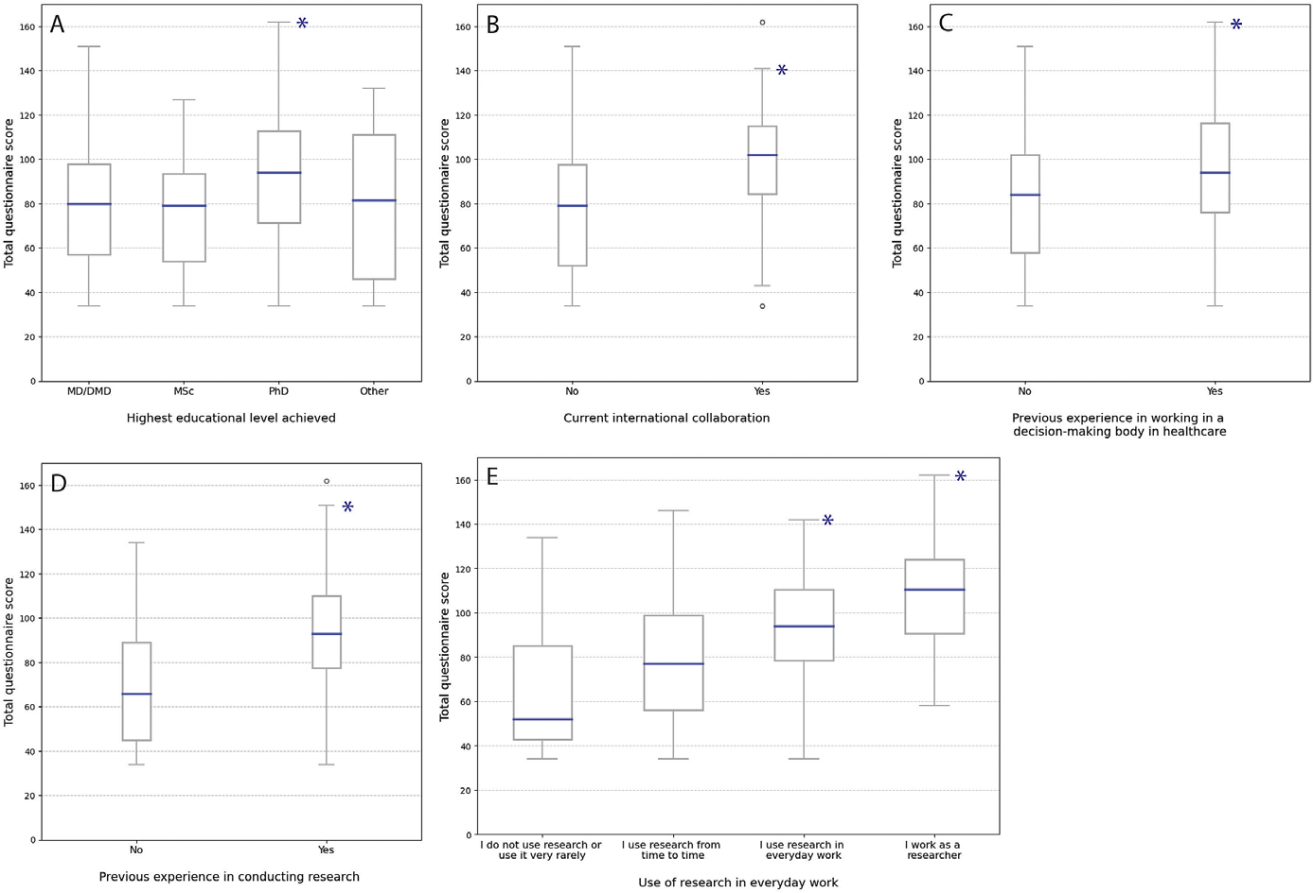
Total questionnaire score for skills required for the HTA process according to demographic characteristics of the respondents. A. Highest education level; asterisk indicates significant difference from the MD/DMD group. B. Current international collaboration; asterisk indicates significant difference between the groups. C. Previous experience in working in a decision-making body in healthcare; asterisk indicates significant difference between the groups. D. Previous experience in conducting research; asterisk indicates significant difference between the groups. E. Use of research in everyday work or being a researcher. Asterisks indicate a statistically significant difference between the groups “I do not use research or use it very rarely” or “I use research from time to time.” Kruskal-Wallis test with Dunn post-hoc analysis. The total questionnaire score ranged from 34 to 170 for 10 sections (Supplementary Table 1).

We analyzed the total HTA-skills scores needed to act as an individual clinical expert or a member of health professional organizations across demographic and professional subgroups (Supplementary Tables 6–12). Respondents with a PhD degree, those engaged in international collaboration, those with experience conducting research, those who had participated in healthcare decision-making bodies, and those who used research regularly in their daily work (“use of research” in this context refers to reading and applying peer-reviewed scientific publications in daily professional work) or identified as researchers, had significantly higher total scores (Figure 1).

Other demographic variables, such as age, hospital of employment, and years of professional experience, showed no statistically significant associations (Supplementary Tables 13–15).

## Discussion

This study examined Croatian doctors’ skills and preparedness levels for participating in HTA processes at both national and European levels. Results indicate significant gaps in HTA skills and experience among Croatian medical/dental doctors, needed to act mainly as individual clinical experts or members of health professional organizations, particularly concerning the HTAR and four areas of joint work.

These findings reflect the fact that the HTA process in Croatia has not laid ground within the decision-making process (30;34). Some medical doctors have been involved in the national HTA process but also in joint HTA processes through EUnetHTA Joint Actions, as individual clinical experts or as representatives from their respective organizations, only on an ad hoc basis, providing their clinical expertise and views mainly related to their disease/therapeutic areas and organizational domain (24;31). Some of them are also involved in pharmaceutical or medical device committees at the Croatian Health Insurance Fund, where they appraise the manufacturer’s submission folders and review national HTA reports, as requested (35;36). The particularly low self-reported familiarity with the HTAR (64.1 percent of respondents indicated they were not at all familiar with it) raises concerns about clinicians’ preparedness to engage with HTAR-related activities in 2025. Specifically, this limited awareness may affect their ability to contribute as individual clinical experts to defining nationally relevant PICO questions for JCAs or to provide input during JSCs. However, this finding reflects the readiness of clinical practitioners in Croatia rather than the overall institutional capacity (HTA doers) of Croatia to perform HTAR-related tasks.

Examining these results within the broader regional context, it is evident that in recent years, many CEE countries have undertaken initiatives to strengthen their HTA capacity and align with European standards. Although significant progress has been made, including the establishment of dedicated HTA agencies, enhanced methodological guidance, and increased participation in European joint assessments, a substantial amount of work remains. Persistent challenges remain in ensuring methodological consistency, sustainable funding, and structured stakeholder involvement. Recent analyses highlight that, despite encouraging developments, CEE countries continue to face barriers related to limited institutional resources, uneven expertise, and the need for stronger integration with EU-level HTA processes (20;37;38).

The assessment of specific HTA process-related skills, needed to act as an individual clinical expert or member of health professional organizations, revealed varying levels of competency, with median scores ranging from 40 percent to 60 percent of the maximum possible scores. The lowest scores were obtained in domains related to health economics, grading the certainty of evidence, qualitative evidence synthesis, and public/patient involvement, where respondents achieved only 40 percent of the maximum score. Advancement in skills related to these scores is essential for possible work as individual clinical experts within different areas of joint work or HTA at the national level, but even more to work as HTA doers, with complete methodological HTA expertise. This finding mirrors challenges identified in other CEE healthcare systems, where economic evaluation skills among healthcare professionals are often insufficient to enable the practical interpretation and application of HTA findings in clinical or policy decision-making (39). Regarding the qualitative evidence synthesis, low scores can also be attributed, among other factors, to structural barriers, including limited institutional support for qualitative research in medical settings and fewer opportunities for hands-on experience with qualitative methods (40). Furthermore, qualitative synthesis requires distinct skills from quantitative analysis, including interpretive approaches, understanding of theoretical frameworks, and methods for synthesizing narrative data, which are not typically part of standard medical training (41). These challenges are particularly evident in medicinal product development, where quantitative methods, especially RCTs, represent the primary source of evidence. On the other hand, low performance in grading the certainty of evidence reflects broader systemic challenges in evidence assessment competencies. Many clinicians, despite being familiar with reading evidence summaries, lack formal training in evidence grading methodologies and systematic approaches to assessing certainty of evidence (42). This gap is particularly pronounced in healthcare systems where systematic review methodology and evidence-based healthcare are still evolving in everyday clinical practice (43). Low scores in public and patient involvement can be explained by the traditionally paternalistic approach to healthcare delivery that has historically characterized many healthcare systems, particularly in CEE countries (44). Despite growing recognition of its importance, meaningful patient involvement in healthcare decision-making remains a relatively new concept in many settings, with limited institutional frameworks and practical experience in implementing patient engagement strategies (45). HTA doers and decision-makers on health technologies often lack training in participatory approaches and methods for incorporating patient perspectives into HTA. In contrast, organizational structures frequently lack established mechanisms for systematic patient involvement (46). Involvement of patient representatives is especially warranted in defining the scope of a JCA and commenting on the JCA draft, the absence of which might jeopardize the usefulness and uptake of JCAs on the national level. Recent international reviews highlight a growing recognition among HTA bodies of the need to systematically integrate patient engagement and patient experience data into assessment processes, reinforcing the importance of structured frameworks that ensure this input meaningfully informs healthcare decision-making (47;48).

When analyzing the differences in skills according to demographic factors, education level emerged as a significant factor in HTA competency, with PhD holders demonstrating higher skill levels across multiple domains. This finding supports previous research, suggesting that advanced academic training contributes to a better understanding and implementation of evidence-based healthcare practices (49). However, the overall low scores, even among highly educated professionals, indicate that traditional academic credentials alone may not be sufficient for the HTA expertise needed in HTA processes and within decision-making processes (50). Also, significantly higher scores across all HTA competency categories among the respondents with active international collaborations align with previous research on the benefits of cross-border professional networks (51). International collaboration exposes healthcare professionals to diverse methodological approaches, different healthcare systems, and varied HTA practices, thereby broadening their skill set and understanding (52). Such collaborations often provide opportunities for knowledge exchange, participation in multinational research projects, and exposure to advanced HTA methodologies that might not be available in their local context (53). Furthermore, international networks facilitate access to expertise and resources to enhance professionals’ capabilities in complex areas such as health economics and evidence synthesis (54). This finding is particularly relevant for healthcare systems in transition, where international collaboration can serve as a crucial mechanism for capacity building and knowledge transfer in the implementation of HTA (55). Building on these demographic and professional factors that have been shown to produce significant differences in HTA skills may inform the design of more targeted and effective capacity-building interventions, particularly in resource-constrained settings where strategic allocation of educational investments is essential.

Several limitations should be considered when interpreting our results. The use of self-reported competency measures may not fully reflect actual skills, and our sample, though drawn from major medical centers, may not be representative of all Croatian healthcare professionals. We acknowledge that including dental medicine doctors may have introduced a degree of heterogeneity, as their direct involvement in HTA processes is generally less frequent compared with other clinical specialities. However, their inclusion reflects the interdisciplinary composition of Croatia’s medical community and aligns with the structure of national professional associations. Additionally, differences in HTA familiarity among various medical fields (e.g., oncology versus internal medicine) are likely and represent an area for further investigation in future research. On the other hand, the varying response rate, which differs from question to question, hinders the comparability of the results within the survey and the study’s power for questions with a lower number of responses. Nevertheless, to the best of our knowledge, this is the first study researching the level of HTA-related skills, needed mainly to act as individual clinical experts or members of health professional organizations but also as HTA doers, among medical doctors in Croatia, and can provide the basis for a successful, efficient, and sustainable national HTA process.

## Conclusions

This study reveals a notable gap in HTA-related skills and experience levels among Croatian clinicians, needed mainly to act as individual clinical experts or members of health professional organizations but also as HTA doers, with the highest level of expertise required, particularly regarding health economics, evidence synthesis, and familiarity with the European HTA Regulation. These findings underscore the importance of structured educational and institutional support for capacity building at both the national and EU levels.

We recommend a multifaceted approach to improving clinician readiness for HTA participation, primarily as an individual clinical expert or a member of a health professional organization. In the short term, national and European training initiatives should be developed to provide targeted instruction on the scoping process (PICO framework), which is essential for both JCA and JSC processes. This instruction should also focus on establishing “guided collaborations” between clinicians and HTA analysts during the PICO development phases and procedural steps of JSC. Medium-term measures could include key concepts such as evidence synthesis and economic evaluation (essential for the national non-clinical domain), as well as other HTAR processes relevant for horizon scanning and various steps of JCA or JSC (e.g., a clinical development plan, including the appropriate study design and post-launch evidence generation). In the longer term, integrating HTA-related competencies into undergraduate and postgraduate medical education will ensure sustained expertise and engagement, mainly to act as individual clinical experts or members of health professional organizations and as the first step in the extended education for HTA doers.

Beyond training, systematic dissemination of HTA results, through professional associations, continuing medical education, and communication of JCA outcomes, should be implemented to raise awareness of the role and relevance of HTA among healthcare professionals. Such activities would also support the goals of the HTAR by fostering informed stakeholder participation and improving the practical uptake of joint assessments at the national level.

By addressing these gaps through education, collaboration, and dissemination, Croatia and similar health systems can strengthen their capacity to contribute effectively to the European HTA ecosystem. This would ensure that clinical perspectives are appropriately reflected in the JCAs and other collaborative activities envisioned under HTAR, ultimately improving evidence-based healthcare decisions at national levels and patient outcomes across the EU.

Finally, the tool used in our study can serve as a valuable addition for tracking progress in developing expertise and skills in Croatia, as well as countries with similar socioeconomic backgrounds, health systems, and educational/research capacities in Europe. As the HTAR is being implemented, these initiatives will be crucial for building the necessary capacity within the healthcare system and ensuring the successful adoption of this new HTA framework.

## Supporting information

10.1017/S0266462326103572.sm001 Vuković et al. supplementary material 1 Vuković et al. supplementary material

10.1017/S0266462326103572.sm002 Vuković et al. supplementary material 2 Vuković et al. supplementary material

## Supplementary material

The supplementary material for this article can be found at http://doi.org/10.1017/S0266462326103572.
