# Supplementary material for: Level of health technology assessment process-related skills among doctors in Croatia: a cross-sectional survey study
Source: Int J Technol Assess Health Care. 2026 Feb 25;42(1):e34. doi: 10.1017/S0266462326103572 (PMC13071844; doi:10.1017/S0266462326103572)
Supplement: Vuković et al. supplementary material 1 — Vuković et al. supplementary material [file S0266462326103572sup001.docx]

**Upitnik za procjenu vještina potrebnih za procjene zdravstvenih tehnologija na nacionalnoj razini ili razini EU**

Poštovani,

Pozivamo Vas da sudjelujete u istraživanju o poznavanju postupka procjene zdravstvenih tehnologija (eng. HTA – *health technology assessment*). Istraživanje se odvija u okviru projekta Hrvatske zaklade za znanost ProDeM – Profesionalizam u zdravstvu: Odlučivanje u praksi i znanosti. Istraživanje je odobrilo Etičko povjerenstvo Medicinskog fakulteta Sveučilišta u Splitu..

Ljubazno Vas molimo da ispunite ovaj upitnik. Naglašavamo da je upitnik potpuno anoniman i dobrovoljan. Elektronički oblik ove ankete ne prikuplja informaciju o Vašoj IP-adresi, tako da je u potpunosti anoniman i ne podliježe odredbama Opće uredbe o zaštiti osobnih podataka (GDPR). Vaše odgovore ćemo obraditi na razini svih prikupljenih anketa, ne pojedinačno, a dobivene rezultate ćemo koristiti isključivo u znanstveno-istraživačke svrhe. Prikupljeni podaci će se koristiti isključivo za potrebe ovog istraživanja i provedbu edukativnih aktivnosti usmjerenih k podizanju spremnosti za sudjelovanje u postupcima HTA nakon početka primjene Uredbe o HTA Europske komisije u siječnju 2025. godine. Upitnik se sastoji od dva dijela: općih informacija o Vama i pitanja u kojima dajete svoje iskustvo ili mišljenje. Dio upitnika o HTA je na engleskom jeziku, kako bismo što bolje prenijeli međunarodnu terminologiju koja će se koristiti u izradi zajedničkih HTA na EU razini. Detaljan prijevod Uredbe na hrvatski jezik dostupan je ovdje: <https://eur-lex.europa.eu/legal-content/HR/TXT/HTML/?uri=CELEX:32021R2282>.

Prosječno vrijeme potrebno za popunjavanje testa je 15-20 minuta. Ispunjavanje testa smatramo Vašim informiranim pristankom za sudjelovanje u istraživanju.

Unaprijed zahvaljujemo na Vašem sudjelovanju.

**POJMOVI**

*(izvor:* *Uredba o HTA 2021/2282)*

**Health Technology Assessment / Procjena zdravstvenih tehnologija**

*„****Procjena zdravstvene tehnologije****” znači multidisciplinaran postupak kojim se sažimaju informacije o medicinskim i socijalnim aspektima te aspektima koji se odnose na pacijente, kao i ekonomskim i etičkim pitanjima povezanima s uporabom pojedine zdravstvene tehnologije na sustavan, transparentan, nepristran i pouzdan način (Uredba 2021/2282, članak 2).*

**Joint Clinical Assessment** / **Zajednička klinička procjena**

*„****Zajednička klinička procjena****” pojedine zdravstvene tehnologije znači znanstveno prikupljanje i opisivanje komparativne analize dostupnih kliničkih dokaza o pojedinoj zdravstvenoj tehnologiji u usporedbi s nekom drugom zdravstvenom tehnologijom ili s više njih ili postojećim postupcima, u skladu s opsegom procjene dogovorenim u okviru ove Uredbe i na temelju znanstvenih aspekata sljedećih kliničkih domena procjene znanstvenih tehnologija: opisa zdravstvenog problema koji se pokušava riješiti zdravstvenom tehnologijom i trenutačne uporabe drugih zdravstvenih tehnologija kojima se pokušava riješiti taj zdravstveni problem, opisa i tehničke karakterizacije zdravstvene tehnologije, relativne kliničke djelotvornosti i relativne sigurnosti zdravstvene tehnologije (Uredba 2021/2282, članak 2).*

**Non-clinical Assessment / Neklinička procjena**

*„****Neklinička procjena****” znači dio procjene zdravstvenih tehnologija koji se temelji na sljedećim nekliničkim domenama procjene zdravstvenih tehnologija: troškovima i ekonomskoj evaluaciji pojedine zdravstvene tehnologije te etičkim, organizacijskim, socijalnim i pravnim aspektima povezanima s njezinom uporabom (Uredba 2021/2282, članak 2).*

**Collaborative Assessment / Suradnička procjena**

*„****Suradnička procjena****” znači klinička procjena medicinskog proizvoda ili in vitro dijagnostičkog medicinskog proizvoda koju na razini Unije provode zainteresirana nadležna i druga tijela za procjenu zdravstvenih tehnologija koja dobrovoljno sudjeluju (Uredba 2021/2282, članak 2).*

**Horizon scanning (emerging health technologies) / Ispitivanje vidokruga (zdravstvene tehnologije u nastajanju)**

***Ispitivanje vidokruga (zdravstvene tehnologije u nastajanju)*** *omogućuje rano utvrđivanje zdravstvenih tehnologija u nastajanju za koje postoji vjerojatnost da će imati velik učinak na pacijente, javno zdravlje i sustave zdravstvene zaštite te se koristiti u istraživanjima. Takvo ispitivanje vidokruga također može pružiti informacije za potrebe dugoročnog planiranja na razini Unije i na nacionalnoj razini (Uredba 2021/2282, točka 42, članak 22).*

**Voluntary cooperation / Dobrovoljna suradnja**

*Komisija podupire suradnju i razmjenu znanstvenih informacija među državama članicama u pogledu:*

*(a) nekliničkih procjena zdravstvenih tehnologija;*

*(b) suradničkih procjena medicinskih proizvoda i in vitro dijagnostičkih medicinskih proizvoda;*

*(c) procjena zdravstvenih tehnologija kad je riječ o zdravstvenim tehnologijama koje nisu lijekovi, medicinski proizvodi ili in vitro dijagnostički medicinski proizvodi;*

*(d) pružanja dodatnih dokaza koji su potrebni za potporu procjenama zdravstvenih tehnologija, posebice u vezi sa zdravstvenim tehnologijama za milosrdnu uporabu i zastarjelim zdravstvenim tehnologijama;*

*(e) kliničkih procjena zdravstvenih tehnologija za koje još nije pokrenuta zajednička klinička procjena i zdravstvenih tehnologija koje nisu navedene u tom članku, posebno zdravstvenih tehnologija za koje je u izvješću o zdravstvenim tehnologijama u nastajanju zaključeno da se očekuje da će imati znatan učinak na pacijente, javno zdravlje ili sustave zdravstvene zaštite (Uredba 2021/2282, točka 43, članak 23).*

**Scientific consultation / Znanstveno savjetovanje**

*Kako bi se olakšao postupak pripreme zajedničkih kliničkih procjena, subjektima koji razvijaju zdravstvenu tehnologiju trebalo bi, u odgovarajućim slučajevima, omogućiti sudjelovanje u zajedničkim znanstvenim savjetovanjima s Koordinacijskom skupinom kako bi dobili smjernice o informacijama, podacima, analizama i drugim dokazima koji će se vjerojatno zahtijevati iz kliničkih studija. Kliničke studije obuhvaćaju klinička ispitivanja lijekova, klinička ispitivanja potrebna za kliničku procjenu medicinskih proizvoda i studije učinkovitosti potrebne za procjene učinkovitosti in vitro dijagnostičkih medicinskih proizvoda. S obzirom na preliminarnu prirodu savjetovanja, ni jedna ponuđena smjernica ne bi smjela biti pravno obvezujuća za subjekte koji razvijaju zdravstvenu tehnologiju ili za nadležna i druga tijela za procjenu zdravstvenih tehnologija. Takve bi smjernice, međutim, trebale odražavati najnovija ostvarenja medicinske znanosti u vrijeme zajedničkog znanstvenog savjetovanja, posebice u interesu pacijenata.* *Ako se zajednička znanstvena savjetovanja provode usporedno s pripremom znanstvenih savjeta o lijekovima predviđenih Uredbom (EZ) br. 726/2004 ili usporedno sa savjetovanjem o medicinskim proizvodima predviđenim Uredbom (EU) 2017/745, ti bi se usporedni postupci, uključujući razmjenu informacija između podskupina i Europske agencije za lijekove ili stručnih skupina za medicinske proizvode, trebali provoditi s ciljem osiguravanja da se prikupljanjem dokaza ispune potrebe dotičnih okvira, pritom zadržavajući odvojenost njihovih odgovarajućih nadležnosti (Uredba 2021/2282, točke 39 i 40, članci 16-21).*

**OPĆE INFORMACIJE**

**1. Dob (u godinama): 2. Spol (zaokružite): 3. Kojim se jezikom služite**

1. Manje od 30 A. Ženski **osim hrvatskim** (npr. prevesti
2. 31-40 B. Muški članak, proučiti podatke)?
3. 41-50
4. 51-60
5. Stariji od 60 godina

**4. Koja je Vaša najveća razina obrazovanja? 5. Koje je Vaše glavno područje profesionalnog interesa**

1. Diplomski studij **(specijalizacija, znanstveno područje)?**
2. Magisterij
3. Doktorat
4. Drugo, molimo navedite:

**6. Koliko imate godina radnog iskustva? 7. Imate li trenutno međunarodnu stručnu ili**

1. <5 **znanstvenu suradnju?**
2. 5-10 A. Da
3. 11-15 B. Ne
4. 16-20
5. Više od 20

**8. Jeste li ikada sudjelovali u radu stručnog povjerenstva ili tijela koje donose odluke (npr. Povjerenstvo za lijekove HZZO-a, Povjerenstvo za medicinske proizvode, Etičko povjerenstvo, član Upravnog vijeća…)?**

1. Da
2. Ne

Ako da, u kojem povjerenstvu i koliko godina ste radili/radite?

**_____________________________________**

**9. Imate li iskustva u provođenju znanstvenih istraživanja?**

1. Da
2. Ne

Ako da, koliko godina?____

**10. Koja od izjava najbolje opisuje Vašu uporabu istraživanja u svakodnevnom radu?**

1. Ne koristim istraživanja ili ih jako rijetko koristim
2. Koristim istraživanja s vremena na vrijeme
3. Koristim istraživanja u svakodnevnom radu
4. Radim kao istraživač

*Istraživanje: U ovom kontekstu istraživanjem smatramo bilo kakvu studiju o konkretnom problemu ili pitanju provedenu korištenjem znanstvene metodologije*

**PITANJA O HTA**

*Pitanja koja slijede su na engleskom jeziku kako bi se sačuvala izvorna terminologija koja će se koristiti u zajedničkim HTA analizama na razini EU.*

**1. Have you ever been involved in the HTA process? 2. Have you ever used the results of an HTA?**

1. Yes A. Yes
2. No B. No

If yes: If yes, for which purpose?

1. At national level
2. At EU level

**3. Have you ever undertaken a systematic review or other type of evidence synthesis (e.g. clinical guideline, mapping review)?**

1. Yes
2. No

3.1 If yes, how many?

1. 1-5
2. 6-10
3. More than 10

| 3.2 If yes, what type of review/guideline/analysis did you work on? (select all that apply) | 3.3 If yes, what best describesd your role(s)? (select all that apply) |
| --- | --- |
| 1. Systematic review (SR) of intervention 2. SR of Diagnostic Tests accuracy 3. SR of prognosis studies 4. Network Meta-Analysis (NMA) 5. Rapid review 6. Scoping/mapping review 7. Clinical guideline 8. SR/Rapid review within Health Technology Assessment (HTA) 9. Other, please specify: | 1. Project lead 2. Senior responsible 3. Stakeholder 4. Project manager 5. Advisor 6. Researcher 7. HTA doer 8. HTA user 9. Health economist 10. One of many collaborators |

**4. Have you ever undertaken an economic evaluation?**

1. Yes
2. No

4.1 If yes, how many?

1. 1-5
2. 6-10
3. More than 10

| 4.2 If yes, what type of economic evaluation did you work on? (select all that apply) | 4.3 If yes, what best describes your role(s)? (select all that apply) |
| --- | --- |
| 1. Cost Effective Analysis (CEA) 2. Cost Benefit Analysis (CBA) 3. Cost Utility Analysis (CUA) 4. Cost Minimisation Analysis (CMA) 5. Cost Consequences Analysis (CCA) 6. Other, please specify: | 1. Project lead 2. Senior responsible 3. Stakeholder 4. Project manager 5. One of many collaborators 6. Advisor 7. Health economist |

**5. Have you ever used the results of an economic evaluation?** Yes / No

If yes, for which purposes?

**Have you ever critically appraised the quality of any of the following? (choose all that apply)**

|  | **Yes** | **No** | **If yes, please add the name of the instrument(s) e.g. Cochrane RoB, CHEERS, ROBINS-I** |
| --- | --- | --- | --- |
| Randomized controlled trial |  |  |  |
| Non-randomized controlled trial |  |  |  |
| Observational study |  |  |  |
| Diagnostic/Prognostic study |  |  |  |
| Qualitative study |  |  |  |
| Systematic review of interventions |  |  |  |
| Economic evaluations |  |  |  |
| Clinical practice guideline |  |  |  |

**Conflict of interest and confidentiality**

Do you understand the concept and importance of potential conflict of interest (e.g., within the health technology assessment process)?

1. Yes
2. No

Do you understand the concept and importance of confidentiality (e.g., within the process of scientific consultations)?

1. Yes
2. No

**SKILLS NEEDED IN THE HTA PROCESS AS A CLINICAL EXPERT/MEMBER OF HEALTH PROFESSIONAL ORGANISATIONS**

**Clinical effectiveness and safety – Please indicate your level of comfort:**

|  | **No knowledge of this topic** | **I have heard of this topic but I don’t feel confident to do it** | **Slightly confident to do it** | **I am confident to do it** | **I have full expertise in this topic** |
| --- | --- | --- | --- | --- | --- |
| Understanding the PICO acronym | 1 | 2 | 3 | 4 | 5 |
| Writing a structured research question according to the PICO acronym | 1 | 2 | 3 | 4 | 5 |
| Formulating the key words for the research question | 1 | 2 | 3 | 4 | 5 |

**Searching for the studies – Please indicate your level of comfort with:**

|  | **No knowledge of this topic** | **I have heard of this topic, but I don’t feel confident to do it** | **Slightly confident to do it** | **I am confident to do it** | **I have full expertise in this topic** |
| --- | --- | --- | --- | --- | --- |
| Understanding the importance of Electronic sources and databases to search for evidence | 1 | 2 | 3 | 4 | 5 |
| Understanding the importance of grey literature: ongoing studies and unpublished data sources | 1 | 2 | 3 | 4 | 5 |
| Understanding the importance of Other search approaches (hand searching and citation snowballing) | 1 | 2 | 3 | 4 | 5 |

**Critical appraisal skills – Please indicate your level of comfort:**

|  | **No knowledge of this topic** | **I have heard of this topic, but I don’t feel confident to do it** | **Slightly confident to do it** | **I am confident to do it** | **I have full expertise in this topic** |
| --- | --- | --- | --- | --- | --- |
| Identifying validated tools to critically appraise scientific literature | 1 | 2 | 3 | 4 | 5 |
| Recognising the most important domains to appraise in the different study designs | 1 | 2 | 3 | 4 | 5 |
| Understanding of the procedure through which critical appraisal should be undertaken | 1 | 2 | 3 | 4 | 5 |

**Summarizing study characteristics and preparing for synthesis – Please indicate your level of comfort:**

|  | **No knowledge of this topic** | **I have heard of this topic, but I don’t feel confident to do it** | **Slightly confident to do it** | **I am confident to do it** | **I have full expertise in this topic** |
| --- | --- | --- | --- | --- | --- |
| Understanding the summary of the characteristics of each study | 1 | 2 | 3 | 4 | 5 |
| Determining comparability across studies (i.e. clinical, statistical, and methodological heterogeneity) | 1 | 2 | 3 | 4 | 5 |
| Understanding the relevant comparisons from the included studies | 1 | 2 | 3 | 4 | 5 |

**Qualitative evidence synthesis (QES) – Please indicate your level of comfort in:**

|  | **No knowledge of this topic** | **I have heard of this topic, but I don’t feel confident to do it** | **Slightly confident to do it** | **I am confident to do it** | **I have full expertise in this topic** |
| --- | --- | --- | --- | --- | --- |
| Understanding the use of QES in HTA | 1 | 2 | 3 | 4 | 5 |
| Understanding the methods for QES: thematic synthesis, framework synthesis, and meta-ethnography | 1 | 2 | 3 | 4 | 5 |
| Understanding the importance of Balanced description and interpretation | 1 | 2 | 3 | 4 | 5 |

**Grading the certainty of the evidence – Please indicate your level of comfort with:**

|  | **No knowledge of this topic** | **I have heard of this topic, but I don’t feel confident to do it** | **Slightly confident to do it** | **I am confident to do it** | **I have full expertise in this topic** |
| --- | --- | --- | --- | --- | --- |
| Understanding the summary of findings (SoF) tables or evidence profiles | 1 | 2 | 3 | 4 | 5 |
| Understanding the ranking of the outcomes for SoF tables or evidence profiles | 1 | 2 | 3 | 4 | 5 |
| Understanding the various approaches for assessing the certainty of a body of evidence | 1 | 2 | 3 | 4 | 5 |

**Understanding key concepts in data synthesis and analysis – Please indicate your level of comfort with:**

|  | **No knowledge of this topic** | **I have heard of this topic, but I don’t feel confident to do it** | **Slightly confident to do it** | **I am confident to do it** | **I have full expertise in this topic** |
| --- | --- | --- | --- | --- | --- |
| Results from meta-analysis | 1 | 2 | 3 | 4 | 5 |
| Results from meta-regression | 1 | 2 | 3 | 4 | 5 |
| Heterogeneity | 1 | 2 | 3 | 4 | 5 |
| Results from subgroup and sensitivity analysis | 1 | 2 | 3 | 4 | 5 |
| Results from narrative synthesis when a meta-analysis is not possible (e.g. Logical categories) | 1 | 2 | 3 | 4 | 5 |
| Results from network meta-analysis | 1 | 2 | 3 | 4 | 5 |
| Principles of a network meta-analysis (transitivity or evidence network) | 1 | 2 | 3 | 4 | 5 |

**Ethics – Please indicate your level of comfort with:**

|  | **No knowledge of this topic** | **I have heard of this topic, but I don’t feel confident to do it** | **Slightly confident to do it** | **I am confident to do it** | **I have full expertise in this topic** |
| --- | --- | --- | --- | --- | --- |
| Understanding the ethical issues concerning technologies | 1 | 2 | 3 | 4 | 5 |
| Understanding the bioethical issues and concepts (self-determination, privacy, informed consent, etc.) | 1 | 2 | 3 | 4 | 5 |

**Public and Patient Involvement (PPI) (adapted from PPEET Tool) – Please indicate your level of comfort with:**

|  | **No knowledge of this topic** | **I have heard of this topic, but I don’t feel confident to do it** | **Slightly confident to do it** | **I am confident to do it** | **I have full expertise in this topic** |
| --- | --- | --- | --- | --- | --- |
| Understanding the importance of Public and patient engagement (PPI) in HTA | 1 | 2 | 3 | 4 | 5 |
| Understanding the Mode of Engagement* | 1 | 2 | 3 | 4 | 5 |
| Understanding the process of identification and recruitment of those most affected by the decision | 1 | 2 | 3 | 4 | 5 |
| Understanding the input from PPI | 1 | 2 | 3 | 4 | 5 |

*****Mode of engagement:

1. Communication = Public & Patient (PP) receive information but have no role in contributing to HTA;

2. Consultation = PP provide their views, thoughts, opinions but there is no commitment to act on them;

3. Collaboration = PPI are engaged to influence the production of the HTA (e.g. commenting, advising, voting;

4. Co-production = PP are equal members of the HTA development team and participate in all steps of the HTA development process

**Health economics (adapted from IDSI's economic tool) – Please indicate your level of comfort with:**

|  | **No knowledge of this topic** | **I have heard of this topic, but I don’t feel confident to do it** | **Slightly confident to do it** | **I am confident to do it** | **I have full expertise in this topic** |
| --- | --- | --- | --- | --- | --- |
| Understanding the objectives of economic evaluation of health interventions, for example using cost-effectiveness analysis, cost-utility analysis | 1 | 2 | 3 | 4 | 5 |
| Understanding the results of economic evaluation of health interventions (cost-effectiveness analysis, cost-utility analysis) | 1 | 2 | 3 | 4 | 5 |
| Understanding the results of budget impact analysis | 1 | 2 | 3 | 4 | 5 |

**knowledge, skills, and experience related to eu joint work**

**(in the context of eu hta regulation)**

**1. How familiar are you with the EU HTA Regulation?**

1. Not at all
2. Heard of it
3. Familiar about details

**2. Do you have previous experience as a clinical expert/member of health professional organisations in joint HTA?** (e.g., within EUnetHTA, with other HTA bodies…)

1. Yes
2. No

If yes, in which part of joint HTA did you provide your clinical expertise the most (circle all that apply):

1. In four clinical domains of assessment
2. Identification of a health problem and current health technology
3. Examination of the technical characteristics of the health technology under assessment
4. Relative clinical effectiveness
5. Relative safety
6. In five non-clinical assessment domains
7. Cost and economic evaluation of a health technology
8. Organisational aspects
9. Patient/social aspects
10. Ethical aspects
11. Legal aspects

**3. Do you have previous experience in joint scientific consultation**?

1. Yes
2. No

If yes:

1. In HTA only
2. In parallel with EMA

**4. Do you have experience in horizon scanning (identification of emerging health technologies)?**

1. Yes
2. No

**5. Are you envisaging being involved as a clinical expert/member of health professional organisations within voluntary collaboration on HTA?**

1. Yes
2. No

**6. Are you involved in the preparatory phase related to EU HTA Regulation?**

1. Yes
2. No

If yes: (check all that apply)

1. Involved in the preparatory phase at the EU level (e.g., part of the Coordination group, its Sub-groups)
2. In the sub-group on joint clinical assessment (JCA) related to pharmaceuticals
3. In the sub-group on joint clinical assessment (JCA) related to medical devices
4. In the sub-group on joint scientific consultation related to pharmaceuticals
5. In the sub-group on joint scientific consultation related to medical devices
6. In the sub-group on horizon scanning related to pharmaceuticals
7. In the sub-group on horizon scanning related to medical devices
8. In the sub-group on methodological and procedure guidance related to pharmaceuticals
9. In the sub-group on methodological and procedure guidance related to medical devices

**7. Are you involved in the preparatory phase at the national level?**

1. Yes
2. No

If yes, please specify in which activities:

**8. Are you aware of any published EUnetHTA21 documents (e.g., methodological/procedure guidance, Template for joint work or Template for stakeholders’ involvement)?**

1. Yes
2. No

**9. Are you involved in any activities related to EU support for HTA?**

1. Yes
2. No

If yes: (check all that apply)

1. In projects related to capacity building of HTA national bodies
2. In projects related to capacity building of patient organisations/patient experts
3. In projects related to methodology research

**FUTURE NEEDS RELATED TO HTA ON THE NATIONAL AND EU LEVEL**

In this section, we are interested to know what core or soft skills you would like to develop further to be appropriately involved in the HTA process as a clinical expert**/**member of health professional organisations. By placing “x”, indicate the order of importance, with the number 1 meaning the most important.

**Searching for studies and synthesis of clinical evidence**

|  | **1.** | **2.** | **3.** | **4.** | **5.** |
| --- | --- | --- | --- | --- | --- |
| Understanding the importance of systematic literature search for the studies |  |  |  |  |  |
| Understanding the results of Data synthesis and analysis |  |  |  |  |  |
| Understanding the results of Network meta-analysis |  |  |  |  |  |
| Understanding the results of Qualitative evidence synthesis (QES) |  |  |  |  |  |
| Understanding the results of Grading the certainty of the evidence |  |  |  |  |  |

**Health economics**

|  | **1.** | **2.** | **3.** |
| --- | --- | --- | --- |
| Understanding the objectives of Economic evaluation of health interventions (cost-effectiveness analysis, cost-utility analysis) |  |  |  |
| Understanding the results of Economic evaluation of health interventions (cost-effectiveness analysis, cost-utility analysis) |  |  |  |
| Understanding the results of Budget impact analysis |  |  |  |

**Organisational aspects**

|  | **1.** | **2.** | **3.** |
| --- | --- | --- | --- |
| Understanding the importance of General organisational issues concerning technologies (e.g., medical devices and procedures) |  |  |  |
| Understanding the importance of Specific issues (e.g., Implementation considerations: Facilities at different healthcare levels, Specialists who perform the procedure, Accessibility, Resources availability, Training, Institutional policy, Quality assurance and monitoring system…) |  |  |  |
| Understanding the assessment elements from EUnetHTA Core HTA Model® 3.0 |  |  |  |

**Ethics**

|  | **1.** | **2.** |
| --- | --- | --- |
| Understanding the importance of General ethical issues concerning technologies |  |  |
| Understanding the Bioethical issues and concepts (self-determination, privacy, informed consent, etc.) |  |  |

**Patient and public involvement (PPI)**

|  | **1.** | **2.** | **3.** | **4.** |
| --- | --- | --- | --- | --- |
| Understanding the importance of Public and patient engagement (PPI) in HTA |  |  |  |  |
| Understanding the Mode of Engagement |  |  |  |  |
| Understanding the process of identification and recruitment those most affected by the decision |  |  |  |  |
| Understanding the input from PPI |  |  |  |  |

**Joint clinical assessment (JCA)**

|  | **1** | **2** | **3** | **4** | **5** | **6** |
| --- | --- | --- | --- | --- | --- | --- |
| General knowledge of joint clinical assessment (e.g., procedure related to Scoping process – PICO; procedure and methodology related to joint clinical assessment) |  |  |  |  |  |  |
| Understanding a PICO Survey |  |  |  |  |  |  |
| Understanding how to provide appropriate input on PICO |  |  |  |  |  |  |
| Understanding the input from patient and clinical experts for PICO consolidation |  |  |  |  |  |  |
| Understanding how to use different Template |  |  |  |  |  |  |
| Understanding the results of joint scientific and summary reports |  |  |  |  |  |  |

**Joint scientific consultations (JSC)**

|  | **1.** | **2.** | **3.** | **4.** | **5.** | **6.** |
| --- | --- | --- | --- | --- | --- | --- |
| General knowledge of joint scientific consultation (e.g., procedure related to PICO; procedure related to involvement of stakeholders) |  |  |  |  |  |  |
| Understanding what is in the Industry submission folder |  |  |  |  |  |  |
| Understanding how to be involved in a meeting with a health technology developer |  |  |  |  |  |  |
| Understanding how to provide appropriate input as clinical experts |  |  |  |  |  |  |
| Understanding how to use Template for JSC |  |  |  |  |  |  |
| Understanding the results of joint scientific recommendation report |  |  |  |  |  |  |

**Identification of emerging health technologies (Horizon Scanning)**

|  | **1.** | **2.** | **3.** | **4.** |
| --- | --- | --- | --- | --- |
| Understanding the results of the reports on emerging health technologies expected to have a major impact on patients, public health or healthcare systems |  |  |  |  |
| Understanding the clinical impact and potential organisational and financial consequences of emerging health technologies for national healthcare systems |  |  |  |  |
| Understanding how to provide information as members of the stakeholder network |  |  |  |  |
| Understanding how I can be contacted as a relevant expert to provide information related to emerging health technologies |  |  |  |  |

**Are there any future needs that we have not covered above? Please specify:**

*Hvala na ispunjavanju ankete!*

*
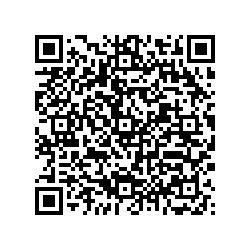
Ako želite doznati rezultate istraživanje ili sudjelovati u daljnjim istraživanjima na ovu temu, možete poslati mail koordinatorima istraživanja skenirajući ovaj QR kod.*
