# Supplementary material for: Level of health technology assessment process-related skills among doctors in Croatia: a cross-sectional survey study
Source: Int J Technol Assess Health Care. 2026 Feb 25;42(1):e34. doi: 10.1017/S0266462326103572 (PMC13071844; doi:10.1017/S0266462326103572)
Supplement: Vuković et al. supplementary material 2 — Vuković et al. supplementary material [file S0266462326103572sup002.docx]

**Supplementary Table 1.** The number of questions and the score range in each section of the questionnaire part “Questions regarding skills needed in the HTA process”

| **Section** | **Number of questions** | **Score range** |
| --- | --- | --- |
| 1. Clinical effectiveness and safety | 3 | 3-15 |
| 1. Searching for the studies | 3 | 3-15 |
| 1. Critical appraisal skills | 3 | 3-15 |
| 1. Summarising study characteristics and preparing for synthesis | 3 | 3-15 |
| 1. Qualitative evidence synthesis | 3 | 3-15 |
| 1. Grading the certainty of evidence | 3 | 3-15 |
| 1. Understanding key concepts in data synthesis and analysis | 7 | 7-35 |
| 1. Ethics | 2 | 2-10 |
| 1. Public and patient involvement | 4 | 4-20 |
| 1. Health economics | 3 | 3-15 |
| **Total** | 34 | 34-170 |

**Supplementary Table 2.** Demographical characteristics of survey respondents*

|  | **N (%)** |
| --- | --- |
| Age (n=376) | |
| <30 | 53 (14.1%) |
| 31-40 | 133 (35.4%) |
| 41-50 | 88 (23.4%) |
| 51-60 | 66 (17.6%) |
| >60 | 36 (9.6%) |
| Gender (n=376) | |
| Male | 131 (34.9%) |
| Female | 245 (65.1%) |
| Highest educational level achieved (n=376) | |
| MD/DMD | 198 (52.7%) |
| MSc | 27 (7.2%) |
| PhD | 130 (34.6%) |
| Other† | 21 (5.5%) |
| Years of working experience (n=375) | |
| <5 | 55(14.7%) |
| 5-10 | 87 (23.2%) |
| 11-15 | 68 (18.1%) |
| 16-20 | 45 (12.0%) |
| >20 | 120 (32.0%) |

MD – Doctor of Medicine; DMD – Doctor of Dental Medicine; MSc – Master of Science, PhD – Doctor of Science

*The number in the bracket next to each item indicates the number of valid responses for each demographic question.

†Includes different subspecialty training.

**Supplementary Table 3. Field of clinical expertise of included participants**

| **Field of expertise** | **N (%)** | **Field of expertise** | **N (%)** |
| --- | --- | --- | --- |
| Anesthesiology | 28 (8.2%) | Psychiatry | 10 (2.9%) |
| Pediatrics | 27 (7.9%) | Endocrinology | 8 (2.4%) |
| Surgery | 25 (7.4%) | Cardiology | 8 (2.4%) |
| Radiology | 25 (7.4%) | Pulmonology | 8 (2.4%) |
| Neurology | 21 (6.2%) | Rehabilitation medicine | 7 (2.1%) |
| Oncology and radiotherapy | 15 (4.4%) | Obstetrics and Gynaecology | 7 (2.1%) |
| Dental medicine | 14 (4.1%) | Emergency medicine | 7 (2.1%) |
| Infectious diseases | 13 (3.8%) | Clinical microbiology | 7 (2.1%) |
| Ophthalmology | 13 (3.8%) | Nuclear medicine | 6 (1.8%) |
| Dermatovenerology | 12 (3.5%) | Orthopaedics and traumatology | 6 (1.8%) |
| Family medicine | 12 (3.5%) | Transfusion medicine | 5 (1.5%) |
| Otorhinolaryngology | 11 (3.2%) | Immunology | 4 (1.2%) |
| Pathology and cytology | 11 (3.2%) | General internal medicine | 4 (1.2%) |
| Public health | 10 (2.9%) | Urology | 3 (0.9%) |
| Other | 13 (3.8%) |  |  |
| Other fields of expertise included: Gastroenterology (2 responses), Genetics (2 responses), Haematology (2 responses), Intensive care (2 responses), Clinical pharmacology (2 responses), Clinical embryology (1 response), Occupational and sports medicine (1 response), and Nephrology (1 response). | | | |

**Supplementary Table 4. Previous experience in evidence synthesis, quality appraisal and economic analysis essential to act as HTA doers**

| What type of review/ guideline/analysis did you work on? | | Have you ever critically appraised the quality of any of the following? | |
| --- | --- | --- | --- |
| SR of intervention | 59 | Randomised controlled trial (n=257) | 72 |
| SR of diagnostic tests' accuracy | 13 | Non-randomised controlled trial (n=257) | 58 |
| SR of prognosis studies | 12 | Observational study (n=257) | 68 |
| Network meta-analysis | 18 | Diagnostic/Prognostic study (n=257) | 45 |
| Rapid review | 26 | Qualitative study (n=256) | 35 |
| Scoping/mapping review | 15 | Systematic review of interventions (n=256) | 58 |
| Clinical guideline | 75 | Economic evaluations (n=257) | 19 |
| SR/Rapid review within HTA | 3 | Clinical practice guideline (n=257) | 56 |
| Other | 3 |  |  |
| None | 252 |  |  |
| What type of economic evaluation did you work on? | | | |
| Cost-Effective Analysis | | 16 | |
| Cost-Benefit Analysis | | 13 | |
| Cost-Utility Analysis | | 5 | |
| Cost Minimisation Analysis | | 2 | |
| Cost Consequences Analysis | | 1 | |
| Other | | 2 | |
| In this section, participants could choose multiple options; therefore, the sum of all numbers does not correspond to the total number of participants. | | | |

**Supplementary Table 5. Results of the internal consistency test**

| **Category** | **Cronbach’s α** | **95% CI** |
| --- | --- | --- |
| Clinical effectiveness and safety | 0.950 | 0.940 – 0.958 |
| Searching for the studies | 0.903 | 0.884 – 0.919 |
| Critical appraisal skills | 0.963 | 0.956 – 0.969 |
| Summarising study characteristics and preparing for synthesis | 0.956 | 0.948 – 0.963 |
| Qualitative evidence synthesis | 0.972 | 0.966 – 0.976 |
| Grading the certainty of evidence | 0.969 | 0.963 – 0.974 |
| Understanding key concepts in data synthesis and analysis | 0.954 | 0.946 – 0.960 |
| Ethics | 0.955 | 0.945 – 0.963 |
| Public and patient involvement | 0.971 | 0.966 – 0.975 |
| Health economics | 0.961 | 0.954 – 0.968 |

95% CI: 95% Confidence Interval

**Supplementary Table 6. Differences in questionnaire results based on previous experience in conducting research**

| **Category** | **Yes** | | **No** | | ***P*** | |  |  |
| --- | --- | --- | --- | --- | --- | --- | --- | --- |
| Clinical effectiveness and safety (n = 272) | | 10.0 (8.0 – 12.0) | | 6.0 (3.25 – 9.0) | | < 0.001 | | |
| Searching for the studies (n = 272) | | 10.0 (8.0 -12.0) | | 7.0 (5.0 – 9.5) | | < 0.001 | | |
| Critical appraisal skills (n = 272) | | 10.0 (8.0 – 12.0) | | 6.0 (3.25 – 9.0) | | < 0.001 | | |
| Summarising study characteristics and preparing for synthesis (n = 272) | | 10.0 (9.0 – 12.0) | | 7.0 (3.0 – 9.0) | | < 0.001 | | |
| Qualitative evidence synthesis (n = 272) | | 6.0 (3.0 – 9.0) | | 3.0 (3.0 – 6.0) | | < 0.001 | | |
| Grading the certainty of evidence (n = 272) | | 8.0 (6.0 – 9.0) | | 4.0 (3.0 – 7.0) | | < 0.001 | | |
| Understanding key concepts in data synthesis and analysis (n = 273) | | 20.0 (15.0 – 24.0) | | 14.0 (8.0 – 19.0) | | < 0.001 | | |
| Ethics (n = 272) | | 7.0 (6.0 – 8.0) | | 6.0 (4.0 – 6.0) | | < 0.001 | | |
| Public and patient involvement (n = 274) | | 8.0 (4.0 – 12.0) | | 6.0 (4.0 – 10.0) | | < 0.001 | | |
| Health economics (n = 274) | | 6.0 (5.0 – 9.0) | | 5.0 (3.0 – 8.0) | | < 0.001 | | |
| Total questionnaire score (n = 268) | | 93.0 (88.7 – 98.4) | | 66.0 (44.8 – 89.8) | | < 0.001 | | |
| *Mann-Whitney test* | | | | | | | |  |

**Supplementary Table 7. Differences in questionnaire results based on current international collaboration**

| **Category** | **No** | **Yes** | ***P*** |
| --- | --- | --- | --- |
| Clinical effectiveness and safety (n = 269) | 6.0 (3.0 – 9.0) | 9.5 (6.0 – 12.0) | < 0.001 |
| Searching for the studies (n = 271) | 9.0 (5.0 – 11.0) | 11.0 (9.0 – 12.0) | < 0.001 |
| Critical appraisal skills (n = 270) | 8.0 (5.0 – 10.0) | 11.0 (9.0 – 12.0) | < 0.001 |
| Summarising study characteristics and preparing for synthesis (n = 270) | 9.0 (5.0 – 11.0) | 11.0 (9.0 – 12.0) | < 0.001 |
| Qualitative evidence synthesis (n = 270) | 4.5 (3.0 – 8.0) | 6.0 (5.0 – 9.0) | 0.002 |
| Grading the certainty of evidence (n = 270) | 6.0 (3.0 – 9.0) | 8.0 (6.0 – 10.0) | < 0.001 |
| Understanding key concepts in data synthesis and analysis (n = 271) | 16.0 (10.0 – 21.0) | 21.0 (16.0 – 25.0) | < 0.001 |
| Ethics (n = 270) | 6.0 (4.0 – 8.0) | 7.0 (6.0 – 8.0) | 0.001 |
| Public and patient involvement (n = 272) | 7.0 (4.0 – 11.0) | 8.0 (6.0 – 14.0) | < 0.001 |
| Health economics (n = 272) | 6.0 (3.0 – 8.0) | 7.0 (6.0 – 9.0) | < 0.001 |
| Total questionnaire score (n = 266) | 79.0 (73.3 – 85.0) | 102.0 (84.3 – 115.0) | < 0.001 |
| *Mann-Whitney test* | | | |

**Supplementary Table 8. Differences in questionnaire results based on previous experience in conducting research**

| **Category** | **Yes** | | **No** | | ***P*** | |  |  |
| --- | --- | --- | --- | --- | --- | --- | --- | --- |
| Clinical effectiveness and safety (n = 269) | | 7.0 (3.0 – 12.0) | | 7.0 (3.0 – 10.0) | | 0.536 | |  |
| Searching for the studies (n = 271) | | 10.0 (7.0 – 12.0) | | 9.0 (6.0 – 11.0) | | 0.039 | |  |
| Critical appraisal skills (n = 270) | | 10.0 (6.0 – 12.0) | | 9.0 (6.0 – 11.0) | | 0.018 | |  |
| Summarising study characteristics and preparing for synthesis (n = 270) | | 10.0 (8.3 – 12.0) | | 9.0 (6.0 – 11.0) | | 0.016 | |  |
| Qualitative evidence synthesis (n = 270) | | 6.0 (3.0 – 8.8) | | 6.0 (3.0 – 8.0) | | 0.129 | |  |
| Grading the certainty of evidence (n = 270) | | 7.0 (3.3 – 10.0) | | 6.0 (3.0 – 9.0) | | 0.089 | |  |
| Understanding key concepts in data synthesis and analysis (n = 271) | | 21.0 (15.0 – 25.0) | | 17.0 (10.5 – 21.0) | | 0.004 | |  |
| Ethics (n = 270) | | 8.0 (6.0 – 8.0) | | 6.0 (4.0 – 8.0) | | < 0.001 | |  |
| Public and patient involvement (n = 272) | | 9.0 (7.0 – 14.8) | | 8.0 (4.0 – 12.0) | | 0.003 | |  |
| Health economics (n = 272) | | 7.0 (6.0 – 11.0) | | 6.0 (3.0 – 8.0) | | 0.002 | |  |
| Total questionnaire score (n = 266) | | 94.0 (83.3 – 107.7) | | 84.0 (58.0 – 102.0) | | 0.007 | |  |
| *Mann-Whitney test* | | | | | | | | |

**Supplementary Table 9. Differences in questionnaire results based on the use of research in everyday work**

| **Category** | **Factor** | **N** | **Average Rank** | **Factor difference** | **Category** | **Factor** | **N** | **Average Rank** | **Factor difference** |
| --- | --- | --- | --- | --- | --- | --- | --- | --- | --- |
| Clinical effectiveness and safety | (2) I use research from time to time | 125 | 124.29 | (3)(4) | Grading the certainty of evidence | (1) I use research from time to time | 125 | 118,31 | (2)(4) |
|  | (3) I use research in everyday work | 87 | 160.02 | (2)(1) |  | (2) I use research in everyday work | 88 | 163,21 | (1)(3) |
|  | (1) I don’t use research or use it very rarely | 37 | 93.84 | (3)(4) |  | (3) I don’t use research or use it very rarely | 37 | 104,07 | (2)(4) |
|  | (4) I work as a researcher | 22 | 178.48 | (1)(2) |  | (4) I work as a researcher | 22 | 187,55 | (1)(3) |
| Searching for the studies | (2) I use research from time to time | 126 | 118.81 | (3)(4) | Understanding key concepts in data synthesis and analysis | (1) I use research from time to time | 125 | 121,98 | (2)(4) |
|  | (3) I use research in everyday work | 88 | 171.30 | (2)(1) |  | (2) I use research in everyday work | 88 | 167,11 | (1)(3) |
|  | (1) I don’t use research or use it very rarely | 37 | 81.85 | (3)(4) |  | (3) I don’t use research or use it very rarely | 38 | 84,88 | (2)(4) |
|  | (4) I work as a researcher | 22 | 196.70 | (2)(1) |  | (4) I work as a researcher | 22 | 191,93 | (1)(3) |
| Critical appraisal skills | (1) I use research from time to time | 125 | 114,12 | (2)(4) | Ethics | (1) I use research from time to time | 124 | 127,07 | (2)(3) |
|  | (2) I use research in everyday work | 88 | 176,29 | (1)(3) |  | (2) I use research in everyday work | 88 | 162,70 | (1)(3) |
|  | (3) I don’t use research or use it very rarely | 37 | 82,09 | (2)(4) |  | (3) I don’t use research or use it very rarely | 38 | 88,87 | (1)(2)(4) |
|  | (4) I work as a researcher | 22 | 196,02 | (1)(3) |  | (4) I work as a researcher | 22 | 167,11 | (3) |
| Summarising study characteristics and preparing for synthesis | (1) I use research from time to time | 125 | 120,57 | (2)(4) | Public and patient involvement | (1) I use research from time to time | 126 | 130,27 | (4) |
|  | (2) I use research in everyday work | 88 | 164,69 | (1)(3) |  | (2) I use research in everyday work | 88 | 153,91 | (3) |
|  | (3) I don’t use research or use it very rarely | 37 | 84,54 | (2)(4) |  | (3) I don’t use research or use it very rarely | 38 | 97,25 | (2)(4) |
|  | (4) I work as a researcher | 22 | 201,64 | (1)(3) |  | (4) I work as a researcher | 22 | 182,75 | (1)(3) |
| Qualitative evidence synthesis | (1) I use research from time to time | 125 | 128,31 | (4) | Health economics | (1) I use research from time to time | 126 | 130,99 | (4) |
|  | (2) I use research in everyday work | 88 | 142,84 | (4) |  | (2) I use research in everyday work | 88 | 146,11 |  |
|  | (3) I don’t use research or use it very rarely | 37 | 112,28 | (4) |  | (3) I don’t use research or use it very rarely | 38 | 111,88 | (4) |
|  | (4) I work as a researcher | 22 | 198,41 | (1)(2)(3) |  | (4) I work as a researcher | 22 | 184,59 | (1)(3) |
| Total questionnaire score | (1) I don’t use research or use it very rarely | 37 | 82,20 | (3)(4) |  |  |  |  |  |
|  | (2) I use research from time to time | 122 | 117,20 | (3)(4) |  |  |  |  |  |
|  | (3) I use research in everyday work | 87 | 167,79 | (1)(2) |  |  |  |  |  |
|  | (4) I work as a researcher | 22 | 198,59 | (1)(2) |  |  |  |  |  |
| *Dunn post hoc test* | | | | | | | | | |

**Supplementary Table 10. Differences in questionnaire results based on the highest educational level achieved**

| **Category** | **Factor** | **N** | **Average Rank** | **Significant difference in the factor** | **Category** | **Factor** | **N** | **Average Rank** | **Significant difference in the factor** |
| --- | --- | --- | --- | --- | --- | --- | --- | --- | --- |
| Clinical effectiveness and safety | (1) MD/DMD | 155 | 125.48 | (2) | Summarising study characteristics and preparing for synthesis | (1) MD/DMD | 155 | 123.56 | (2) |
|  | (2) PhD | 92 | 157.20 | (1) |  | (2) PhD | 92 | 163.35 | (1) |
|  | (3) Other | 12 | 97.88 |  |  | (3) Other | 12 | 129.04 |  |
|  | (4) MSc | 12 | 147.50 |  |  | (4) MSc | 13 | 107.62 |  |
| Searching for the studies | (1) MD/DMD | 156 | 120.07 | (2) | Grading the certainty of evidence | (1) MD/DMD | 155 | 128.18 | (2) |
|  | (2) PhD | 92 | 170.40 | (1)(4) |  | (2) PhD | 92 | 158.21 | (1) |
|  | (3) Other | 12 | 137.04 |  |  | (3) Other | 12 | 106.79 |  |
|  | (4) MSc | 13 | 103.77 | (2) |  | (4) MSc | 13 | 109.50 |  |
| Critical appraisal skills | (1) MD/DMD | 156 | 121.29 | (2) | Understanding key concepts in data synthesis and analysis | (1) MD/DMD | 156 | 126.28 | (2) |
|  | (2) PhD | 92 | 167.06 | (1) |  | (2) PhD | 92 | 157.37 | (1) |
|  | (3) Other | 12 | 131.79 |  |  | (3) Other | 12 | 136.00 |  |
|  | (4) MSc | 12 | 104.71 |  |  | (4) MSc | 13 | 122.38 |  |
| Ethics | (1) MD/DMD | 155 | 122.41 | (2) | Public and patient involvement | (1) MD/DMD | 157 | 125.01 | (2) |
|  | (2) PhD | 92 | 160.90 | (1) |  | (2) PhD | 92 | 152.50 | (1) |
|  | (3) Other | 12 | 153.37 |  |  | (3) Other | 12 | 172.75 |  |
|  | (4) MSc | 13 | 116.23 |  |  | (4) MSc | 13 | 149.65 |  |
| Total questionnaire score | (1) MD/DMD | 153 | 120,74 | (3) |  |  |  |  |  |
|  | (2) MSc | 11 | 117,23 |  |  |  |  |  |  |
|  | (3) PhD | 92 | 159,92 | (1) |  |  |  |  |  |
|  | (4) Other | 12 | 130,96 |  |  |  |  |  |  |
| *Dunn post hoc test* | | | | | | | | | |

**Supplementary Table 11. Differences in questionnaire results based on different uses of research in everyday work**

| **Category** | **1** | **2** | **3** | **4** | ***P*** |
| --- | --- | --- | --- | --- | --- |
| Clinical effectiveness and safety (n = 271) | 3.0  (3.0 – 8.0) | 6.0  (3.0 – 9.0) | 9.0  (6.0 – 12.0) | 11.0  (5.0 – 12.0) | < 0.001 |
| Searching for the studies (n = 273) | 5.0  (3.8 – 9.0) | 9.0  (6.0 – 10.0) | 11.0  (8.0 – 12.0) | 12.0  (9.0 – 13.0) | < 0.001 |
| Critical appraisal skills (n = 272) | 5.0  (3.0 – 9.0) | 8.0  (6.0 – 9.0) | 10.0  (9.0 – 12.0) | 11.0  (10.0 – 12.0) | < 0.001 |
| Summarising study characteristics and preparing for synthesis (n = 272) | 6.0  (3.0 – 9.0) | 9.0  (5.0 – 10.0) | 10.0  (9.0 – 12.0) | 12.0  (10.0 – 12.0) | < 0.001 |
| Qualitative evidence synthesis (n = 272) | 3.0  (3.0 – 7.0) | 5.0  (3.0 – 7.0) | 6.0  (3.0 – 7.5) | 8.5  (6.0 – 11.0) | < 0.001 |
| Grading the certainty of evidence (n = 272) | 4.0  (3.0 – 8.0) | 6.0  (3.0 – 8.0) | 8.0  (6.0 – 9.5) | 9.0  (6.0 – 12.0) | < 0.001 |
| Understanding key concepts in data synthesis and analysis (n = 273) | 10.5  (7.0 – 18.0) | 15.0  (10.0 – 21.0) | 20.5  (15.5 – 24.0) | 22.0  (18.0 – 26.0) | < 0.001 |
| Ethics (n = 272) | 5.5  (2.0 – 6.0) | 6.0  (4.0 – 8.0) | 7.0  (6.0 – 8.0) | 7.0  (6.0 – 8.0) | < 0.001 |
| Public and patient involvement (n = 274) | 4.0  (4.0 – 8.0) | 7.0  (4.0 – 12.0) | 8.0  (4.5 – 12.0) | 12.0  (8.0 – 14.0) | < 0.001 |
| Health economics (n = 274) | 5.0  (3.0 – 8.0) | 6.0  (3.0 – 9.0) | 6.0  (5.0 – 9.0) | 8.5  (6.0 – 11.0) | 0.003 |
| Total questionnaire score (n = 268) | 52.0  (42.5 – 85.3) | 77.0  (56.0 – 99.0) | 94.0  (78.3 – 110.8) | 110.5  (89.0 – 125.0) | < 0.001 |
| *Kruskal-Wallis test;* 1 – I don’t use research or use it very rarely; 2 – I use research from time to time; 3 – I use research in everyday work; 4 – I work as a researcher | | | | | |

**Supplementary Table 12. Differences in questionnaire results based on different levels of education achieved**

| **Category** | **MD/DMD** | **MSc** | **PhD** | **Other** | ***P*** |
| --- | --- | --- | --- | --- | --- |
| Clinical effectiveness and safety (n=271) | 6.0  (3.0 – 10.0) | 8.5  (4.5 – 11.0) | 9.0  (5.5 – 12.0) | 3.5  (3.0 – 6.5) | 0.004 |
| Searching for the studies (n=273) | 9.0  (5.0 – 10.0) | 7.0  (5.8 – 9.0) | 11.0  (8.0 – 12.0) | 10.0  (4.0 – 11.0) | < 0.001 |
| Critical appraisal skills (n=272) | 8.5  (5.0 – 10.0) | 6.0  (4.5 – 9.0) | 10.0  (7.5 – 12.0) | 9.5  (3.0 – 11.) | < 0.001 |
| Summarising study characteristics and preparing for synthesis (n=272) | 9.0  (6.0 – 10.0) | 8.0  (3.0 – 9.5) | 10.0  (8.0 – 12.0) | 9.0  (3.0 – 12.0) | < 0.001 |
| Qualitative evidence synthesis (n=272) | 5.0  (3.0 – 7.0) | 6.0  (3.0 – 6.0) | 6.0  (3.0 – 9.0) | 4.5  (3.0 – 7.5) | 0.068 |
| Grading the certainty of evidence (n=272) | 6.0  (3.0 – 9.0) | 6.0  (3.0 – 8.0) | 8.0  (4.0 – 10.0) | 4.5  (3.0 – 7.0) | 0.006 |
| Understanding key concepts in data synthesis and analysis (n=273) | 16.5  (11.0 – 21.0) | 15.0  (8.5 – 23.0) | 20.5  (14.0 – 25.0) | 19.5  (7.0 – 23.0) | 0.023 |
| Ethics (n=272) | 6.0  (4.0 – 8.0) | 6.0  (4.8 – 6.5) | 7.0  (6.0 – 8.0) | 7.5  (5.0 – 8.0) | 0.001 |
| Public and patient involvement (n=274) | 7.0  (4.0 – 10.0) | 8.0  (4.0 – 13.0) | 8.0  (4.0 – 12.5) | 11.0  (6.0 – 14.5) | 0.016 |
| Health economics (n=274) | 6.0  (3.0 – 8.0) | 6.0  (3.8 – 12.0) | 6.0  (5.0 – 9.0) | 6.0  (3.0 – 9.5) | 0.112 |
| Total questionnaire score (n=268) | 80.0  (56.8 – 98.3) | 79.0  (50.0 – 94.3) | 94.0  (71.0 – 113.5) | 81.5  (45.0 – 112.5) | 0.002 |

**Supplementary Table 13. Category-specific and total score results depending on the age group**

| **Category** | **Less than 30** | **31-40** | **41-50** | **51-60** | **More than 60** | ***P*** | |
| --- | --- | --- | --- | --- | --- | --- | --- |
| Clinical effectiveness and safety (n = 271) | 9.0 (3.8 – 11.3) | 7.0 (3.0 – 11.0) | 6.0 (3.0 – 9.0) | 6.0 (3.8 – 10.5) | 5.0 (3.0 – 10.0) | | 0.180 |
| Searching for the studies (n = 273) | 9.0 (6.0 – 10.0) | 9.0 (6.0 – 11.8) | 9.0 (6.0 – 11.0) | 9.0 (4.0 – 12.0) | 9.0 (4.0 – 11.0) | | 0.429 |
| Critical appraisal skills (n = 272) | 8.0 (6.0 – 9.0) | 9.0 (6.0 – 11.0) | 9.0 (5.8 – 10.3) | 9.0 (4.0 – 12.0) | 9.0 (5.0 – 12.0) | | 0.547 |
| Summarising study characteristics and preparing for synthesis (n = 272) | 9.0 (7.0 – 11.0) | 9.0 (7.0 – 12.0) | 9.0 (5.0 – 10.5) | 9.0 (5.0 – 12.0) | 10.0 (6.0 – 12.0) | | 0.429 |
| Qualitative evidence synthesis (n = 272) | 4.0 (3.0 – 8.0) | 6.0 (3.0 – 8.0) | 6.0 (3.0 – 7.0) | 4.0 (3.0 – 6.0) | 6.0 (3.0 – 9.0) | | 0.252 |
| Grading the certainty of evidence (n = 272) | 6.0 (3.0 – 9.0) | 6.0 (3.3 – 9.0) | 6.0 (3.0 – 8.5) | 4.5 (3.0 – 9.0) | 8.0 (3.0 – 12.0) | | 0.194 |
| Understanding key concepts in data synthesis and analysis (n = 273) | 17.0 (12.0 – 22.0) | 18.0 (14.0 – 23.0) | 15.5 (9.55 – 21.0) | 19.0 (10.0 – 24.0) | 20.0 (10.0 – 25.0) | | 0.326 |
| Ethics (n = 272) | 6.0 (4.0 – 8.0) | 6.0 (5.0 – 8.0) | 6.0 (5.0 – 8.0) | 7.0 (6.0 – 8.0) | 7.0 (5.0 – 8.0) | | 0.487 |
| Public and patient involvement (n = 274) | 6.5 (4.0 – 11.0) | 8.0 (4.0 – 12.0) | 8.0 (4.0 – 12.0) | 7.0 (4.0 – 12.0) | 9.5 (4.0 – 16.0) | | 0.316 |
| Health economics (n = 274) | 6.5 (3.0 – 9.0) | 6.0 (3.0 – 9.0) | 6.0 (3.0 – 7.3) | 6.0 (3.0 – 8.0) | 7.0 (4.0 – 11.0) | | 0.567 |
| Total questionnaire score (n = 268) | 80.5 (64.5 – 100.5) | 88.0 (67.0 – 106.0) | 84.0 (51.0 – 95.0) | 84.0 (49.8 – 115.0) | 94.0 (54.8 – 124.5) | | 0.496 |
| *Kruskal – Wallis test* | | | | | | | |

**Supplementary Table 14. Category-specific and total score results depend on the working experience group**

| **Category** | **<5** | **5-10** | **11-15** | **16-20** | **More than 20** | ***P*** |
| --- | --- | --- | --- | --- | --- | --- |
| Clinical effectiveness and safety (n = 270) | 8.0 (3.0 – 11.0) | 8.0 (3.0 – 11.0) | 7.0 (3.0 – 10.8) | 6.0 (3.0 – 9.0) | 6.0 (3.0 – 10.3) | 0.728 |
| Searching for the studies (n = 272) | 8.5 (6.0 – 10.0) | 9.0 (6.0 – 11.3) | 9.0 (6.3 – 11.0) | 9.0 (6.0 – 11.0) | 10.0 (4.0 – 12.0) | 0.184 |
| Critical appraisal skills (n = 271) | 8.5 (6.0 – 9.0) | 9.0 (6.0 – 11.0) | 9.0 (6.0 – 11.8) | 9.0 (5.0 – 10.0) | 9.0 (5.0 – 12.0) | 0.471 |
| Summarising study characteristics and preparing for synthesis (n = 271) | 9.0 (6.0 – 10.0) | 10.0 (7.0 – 11.0) | 9.0 (7.3 – 12.0) | 9.0 (5.0 – 10.0) | 9.0 (5.0 – 12.0) | 0.323 |
| Qualitative evidence synthesis (n = 271) | 4.0 (3.0 – 6.5) | 6.0 (3.0 – 7.0) | 6.0 (3.0 – 8.8) | 3.0 (3.0 – 6.0) | 6.0 (3.0 – 9.0) | 0.052 |
| Grading the certainty of evidence (n = 271) | 6.0 (3.0 – 8.0) | 6.0 (3.5 – 9.0) | 7.0 (6.0 – 9.0) | 5.0 (3.0 – 6.0) | 6.5 (3.0 – 10.0) | 0.085 |
| Understanding key concepts in data synthesis and analysis (n = 272) | 17.0 (11.5 – 21.0) | 18.0 (14.0 – 23.0) | 19.0 (14.0 – 22.0) | 15.0 (9.0 – 21.0) | 18.0 (9.3 – 24.0) | 0.318 |
| Ethics (n = 271) | 6.0 (4.0 - 7.5) | 6.0 (4.3 – 8.0) | 6.0 (6.0 – 8.0) | 6.0 (5.0 – 7.0) | 7.0 (6.0 – 8.0) | 0.224 |
| Public and patient involvement (n = 273) | 6.0 (4.0 – 11.0) | 8.0 (4.0 – 12.0) | 8.0 (5.0 – 10.0) | 6.5 (4.0 – 8.0) | 8.0 (4.0 – 15.5) | 0.387 |
| Health economics (n = 273) | 6.0 (3.0 – 9.0) | 6.0 (3.0 – 9.0) | 6.0 (3.3 – 8.0) | 6.0 (3.0 – 8.0) | 6.0 (3.0 – 9.0) | 0.910 |
| Total questionnaire score (n = 267) | 79.0 (63.0 – 98.5) | 88.0 (70.0 – 104.0) | 92.0 (66.3 – 102.0) | 79.0 (52.0 – 92.0) | 85.0 (50.5 – 117.8) | 0.288 |
| *Kruskal-Wallis test* | | | | | | |

**Supplementary Table 15. Category-specific and total score results depend on the institution of origin**

| **Category** | **CMA** | **UHC Split** | **UHC Zagreb** | ***P*** |
| --- | --- | --- | --- | --- |
| Clinical effectiveness and safety (n=271) | 6.0 (3.0 – 10.0) | 7.0 (3.0 – 11.0) | 6.0 (3.0 – 12.0) | 0.813 |
| Searching for the studies (n=273) | 8.0 (6.0 – 11.0) | 9.0 (6.0 – 11.0) | 10.0 (7.0 – 12.0) | 0.026 |
| Critical appraisal skills (n=272) | 9.0 (4.0 – 11.0) | 9.0 (6.0 – 10.0) | 10.0 (6.0 – 12.0) | 0.169 |
| Summarising study characteristics and preparing for synthesis (n=272) | 9.0 (4.0 – 10.8) | 9.0 (6.0 – 11.0) | 9.0 (7.0 – 12.0) | 0.215 |
| Qualitative evidence synthesis (n=272) | 6.0 (3.0 – 6.8) | 6.0 (3.0 – 8.0) | 6.0 (3.0 – 6.8) | 0.522 |
| Grading the certainty of evidence (n=272) | 6.0 (3.0 – 9.0) | 6.0 (3.0 – 9.0) | 7.0 (3.0 – 9.8) | 0.371 |
| Understanding key concepts in data synthesis and analysis (n=273) | 17.0 (9.0 – 21.0) | 18.0 (13.0 – 22.0) | 16.0 (11.3 – 25.0) | 0.388 |
| Ethics (n=272) | 6.0 (4.0 – 8.0) | 6.0 (5.0 – 8.0) | 7.5 (6.0 – 8.0) | 0.235 |
| Public and patient involvement (n=274) | 8.0 (4.0 – 14.0) | 8.0 (4.0 – 11.0) | 8.0 (4.0 – 12.0) | 0.155 |
| Health economics (n=274) | 6.0 (3.0 – 9.0) | 6.0 (3.0 – 9.0) | 6.0 (3.0 – 9.0) | 0.936 |
| Total questionnaire score (n=268) | 83.0 (51.3 – 103.3) | 86.0 (64.5 – 102.8) | 88.0 (65.0 – 116.0) | 0.534 |
| *Kruskal–Wallis test; CMA – Croatian Medical Association* | | | | |
